# Evaluation of Sperm DNA Fragmentation in Oligoasthenoteratozoospermia Patients Using Two Different Techniques: TUNEL and Sperm Chromatin Dispersion Assays

**DOI:** 10.31661/gmj.v13i.3515

**Published:** 2024-10-08

**Authors:** Raziye Chegini, Mahshad Khodarahmian, Niloufar Ahmadian, Sadegh Shirian, Farnaz Khadivi, Shahrzad Zhaeentan, Maryam Salem, Narjes Feizollahi, Azim Hedayatpour, Mehdi Abbasi

**Affiliations:** ^1^ Department of Anatomy, School of Medicine, Tehran University of Medical Sciences, Tehran, Iran; ^2^ Department of Urology, faculty of Medicine, Tehran University of Medical Sciences, Tehran, Iran; ^3^ Department of Pathobiology, School of Veterinary Medicine, Shahrekord University, Shahrekord, Iran; ^4^ Shiraz Molecular Pathology Research Center, Dr Daneshbod Path Lab, Shiraz, Iran; ^5^ Medical Plants Research Center, Basic Health Sciences Institute, Shahrekord University of Medical Sciences, Shahrekord, Iran

**Keywords:** DNA Fragmentation, ligoasthenoteratozoospermia, Sperm Chromatin Dispersion, Terminal Deoxynucleotidyl Transferase dUTP Nick End Labeling, Specificity, Sensitivity

## Abstract

Background: Oligoasthenoteratozoospermia (OAT) is the most prevalent male infertility condition that is mainly caused by sperm DNA fragmentation (SDF). This study compared the sensitivity and effectiveness of two different approaches for analyzing SDF in patients with OAT: sperm chromatin dispersion (SCD) and terminal deoxynucleotidyl transferase dUTP nick end labeling (TUNEL). Materials and Methods: In this study, which received ethical committee approval, participants were divided in to normal and OAT groups (n=20 for each). both TUNEL and SCD assays were used to analyze the sperm DNA fragmentation. And Malondialdehyde (MDA) levels was measured to determine levels of lipid peroxidation in the seminal plasma.Results: The TUNEL assay showed better ability to predict OAT patients than that of the SCD. For our patient population, the projected cut-off points for the DNA fragmentation index of 29% and 19% were reported using the TUNEL and SCD tests, respectively. Seminal levels of MDA were significantly higher in the OAT group (P=0.002) than that of control group. Conclusion: OAT patients showed higher MDA levels of seminal plasma and DNA fragmentation than the control group. Although sperm DNA fragmentation can be detected with high efficiency and sensitivity using both TUNEL and SCD assays, the TUNEL test was found to be a more accurate predictor for OAT patients.

## Introduction

The failure of a couple to conceive after a year of trying is known as clinical infertility. About 50% of infertility are related to the male partner conditions [1]. It is commonly known that sexual and fertility dysfunction are the results of the majority of male infertility issues that are linked to both qualitative and quantitative spermatogenesis defects [2]. The most prevalent male infertility disorder is known as oligoasthenoteratozoospermia (OAT), and it is typified by aberrant sperm morphology, reduced sperm motility, and uncommonly mature sperm [3][4]. Sperm DNA fragmentation (SDF) is an important underlying etiology of OAT, in addition to the many known causes, which include aging, varicocele, cryptorchidism, infection, systemic illnesses, testicular trauma, blockages, endocrine disorders, immunological factors, and idiopathic factors [5]. The consistency of sperm Reproductive function requires DNA to be maintained [6].

Research indicates that lipid peroxidation, apoptosis, low sperm quality, and damage to proteins and DNA can result from sperm the harm that reactive oxygen species cause (ROS) [7][8] .Elevated fragmentation of the sperm nucleus has been directly linked to a higher chance of miscarriage, low-quality embryos, and unsuccessful implantation [9]. Damaged sperm can still fertilize eggs, but there may be problems with embryo development [10].

Single- or double-stranded DNA fragmentation is the most frequent kind of DNA fragmentation in the sperm nucleus. [11]. SDF detection has several advantages over conventional semen analysis, including high stability in examination results, accuracy in predicting pregnancy outcome or assisted reproductive results, and the ability to accurately evaluate fertilization ability [12].

Recent years have seen a lot of research on SDF, and while opinions on the most appropriate way to diagnose it are still divided, it seems widely accepted that the degree of sperm nucleus fragmentation is a good predictor of successful reproduction. Therefore, SDF detection is becoming more and more significant in reproductive laboratories as a significant addition to traditional semen analysis. Elevated levels of SDF have been linked to recurrent failures in assisted reproductive technologies [13][14][15]. The World Health Organization (WHO) manual also notes that SDF is a valuable addition and a promising biomarker in the work-up of male infertility; however, it does not offer a clinical context, suggest which tests are most sensitive, or specify diagnostic cut-off values [13]. As a result, various SDF and chromatin condensation measurement techniques have been created. The comet, the sperm chromatin structure assays (SCSA), the TUNEL, and the sperm chromatin dispersion (SCD) are among the most often used tests for SDF (12). Few studies have fully established the clinical utility and interrelationships of these methods, despite the introduction of numerous experiments with various techniques to assess sperm DNA damage. [16]. The SCD test is based on the theory that even after nuclear protein removal and acid denaturation, sperm with DNA fragmentation are unable to generate a halo of scattered DNA loops. [17][18]. One of the most popular methods for assessing SDF is the TUNEL assay. By labeling only the 30 OH terminal with terminal deoxynucleotidyl transferase (TdT), this test measures the incorporation of fluoresceinated dUTP into double- strand DNA breaks (DSBs) or single-strand breaks (SSBs) in DNA that contains free 30 OH extremities[6]. The most common test for assessing SDF in spermatozoa and various endpoint circumstances in assisted and natural reproduction is the TUNEL assay [19]. While the use of different methods for examining SDF has been extensively reviewed, A handful of studies have thoroughly examined the medical value and interactions of the most widely used techniques. Even in large meta-analyses, the inclusion of studies using various SDF assays poses a challenge to reaching firm conclusions[20]. Thus, in this study, we determine seminal plasma MDA, DNA fragmentation. also, determine the sensitivity, specificity, and correlation of two commonly used methods, including TUNEL and SCD, for determining SDF in OAT patients.

## Materials and Methods

**Figure-1:**
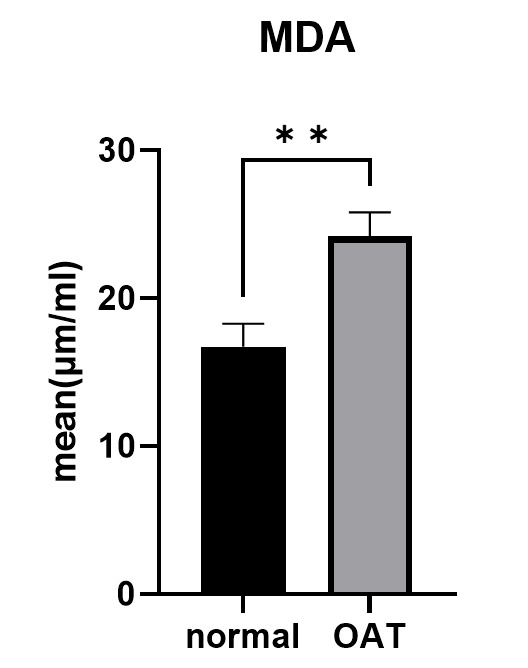
Figure 1. The MDA levels in the OAT and the normal groups were compared. Seminal level of MDA was significantly higher in the OAT group (**: P=0.002), than that of the control group.

**Figure-2:**
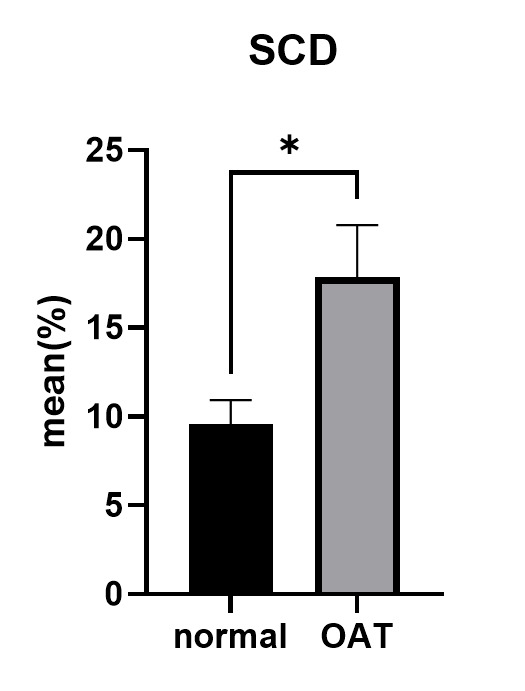
Figure 2. The SCD levels of the OAT group and the normal group were compared. The SCD as significantly increased in the OAT group. *: P=0.015 compared to the normal group.

**Table:** Table1. Sperm Parameters, MDA and SDF in OAT and Normozoospermia

	**NL (n=20) **	**OAT (n=20) **	**P-value**
	Mean ± SD	Mean ± SD	
**Volume (ml) **	2.75±1.03	2.82±1.16	0.679
**Concentration (**million/mL **)**	30.5±9.33	5.9±4.28	<0.001
**Progressive Motility (%) **	47.65±17.91	1.75±3.82	<0.001
**Morphology ** **(%)**	4.1±0.31	1.2±0.83	<0.001
**MDA (%) **	16.73±6.92	24.2±7.15	0.002
**SCD (%) **	9.59±6.01	17.85±13.11	0.015
**TUNEL (%) **	22.02±7.46	33.3±14.56	0.004

Data presented as Mean ± SD. **MDA:** malondialdehyde; **SCD:** sperm chromatin dispersion; **TUNEL:** terminal deoxynucleotidyl transferase dUTP nick end labelling

**Table:** Table2. Comparison of seminal plasma MDA, Semen analysis and Sperm DNA fragmentation between two groups

	**MDA**	**Volume**	**Concentration**	**Progressive motility**	**Morphology**
**MDA**	1	-0.066(0.686)	-0.026(0.872)	0.038(0.818)	-0.035(0.832)
**TUNEL**	0.072(0.658)	-0.065(0.691)	**-0.335(0.034)**	-0.516(0.001)	-0.503(0.001)
**SCD**	0.232(0.149)	-0.098(0.547)	-0.27(0.092)	**-0.367** **(0.020)**	-0.411(0.009)

**MDA:** malondialdehyde; **SCD:** sperm chromatin dispersion; **TUNEL:** terminal deoxynucleotidyl transferase dUTP nick end labelling , coefficient correlation (P-value)

1. Patients’ Selection

The institutional review board of Tehran University of Medical Science gave permission to this study (IR.TUMS.MEDICINE.REC.1402.180). The OAT patients who referred to Arash Women Hospital during June and September of 2023. In this study, 20 samples were considered for the normal group and 20 samples for the case group using G*Power statistical software (version 1.3 Franz Faul, Universitate Kiel, Germany), Twenty normozoospermic men with mean age of 34.8 years old, with an age range of 25 to 40 years old, were also included. None of the participants used alcohol, tobacco, vitamin supplements, or drugs. The study’s objectives were communicated to the participants, who also signed an informed consent form. The semen analysis was conducted in compliance with WHO 2020 guidelines.

2. Sample of Semen

Masturbation was used to obtain the semen samples after three to five days without having sex. The semen samples were incubated at 37°C to complete liquefaction. The computer-assisted semen analysis system Lens Hooke X1PRO® (Bonraybio Co.) was used for the determination of the semen volume, concentration, motility (total, progressive, and non-progressive), pH, and normal morphology by analyzing 40 µL of the sample. The residual semen samples were utilized to analyze sperm lipid peroxidation by measuring the MDA levels and SDF using TUNEL and SCD assays.

3.Semen Analysis

Semen analysis was conducted in compliance with WHO 2020 guidelines (total Sperm concentration ≤20 million/mL; Sperm total motility ≤ 42% or ≤30% Progressive motility; total ejaculate volume 1.0 ml; normal sperm morphology≤4%. Sperm morphology from Diff-Quick stained smears was also conducted according to the criteria of WHO (2020). A total of 200 spermatozoa were observed from each semen specimen. All the smears were evaluated by the same individual.

4. Malondialdehyde (MDA) Assessment

MDA level of seminal plasma was measured by ZellBio GmbH MDA kit (Cat. no. ZBMDA-96A, Germeny). Briefly, MDA testing solution (50 μl) was combined with 50 μl of seminal plasma or standard dilutions. Following that, the mixtures were heated in a bath of boiling water for one hour at 100 °C. After allowing to cool to 37°C, the combined solution was centrifuged at 3000-4000 rpm for 10 min. After isolating the supernatant, spectrometry was used to measure absorbance at 535 nm. Using a standard curve, the quantity of the seminal MDA level was determined.

5. Sperm DNA Fragmentation

TUNEL and SCD assays were used for evaluation of DFITUNEL Assay

5.1. TUNEL Assay

Kit (Roche, Germany) was utilized in compliance with the manufacturer’s instructions to assess sperm DNA damage in the semen sample. Briefly, the semen samples were washed by phosphate-buffered saline (PBS) (Gibco, Germany) twice and prepared smears. The prepared smears were fixed through fixative Buffer Polyformaldehyde dissolved in PBS with a final concentration of 4%. PBS was used to wash the slides three times with 5 min intervals at 37°C. After adding the proteinase K working solution. The slides were then washed with PBS for 3 times. Next, each slide received 100 μL of TdT equilibration working buffer, and it was incubated at room temperature for 30 minutes. Slides were covered with 50 μL of TdT enzyme working solution and incubated for 60 minutes at 37°C in a wet environment. The slides were washed with PBS for 3 times and stained with 4′,6-diamidino-2-phenylindole (DAPI) staining and incubated at 37°C for 5 min. The slides were washed with PBS 4 times. A fluorescence microscope was used to count about 200 spermatozoa. (Olympus BX50, Optica, Olympus DP72). To demonstrate TUNEL’s objectivity and accuracy, positive and negative controls ought to be set up. Sperm of the positive control group were incubated with 100 μL of DNase I solution (Sigma, Germany) and incubated at (25~37°C) for 10-30 min.

5.2. SCD Assessment

The SCD kit was used to assess sperm DNA damage following the guidelines provided by the manufacturer (Idea Venture for the Future, Iran). Briefly, Eppendorf tubes of low-melting-point agarose were heated to 90 to 100 degrees Celsius in a water bath for five minutes. Simultaneously, 50 µl of semen sample with a concentration of 5-10×106 mL of sperm sample was mixed with 1% agarose at 37°C and placed on a slide covered with 65% agarose were obtained from the semen samples, which had been twice cleaned with PBS (Gibco, Germany). Subsequently, a lamella measuring 22 by 22 mm was placed on the slide and maintained at 4°C for five minutes. After removing the lamella, the hydrochloric acid-containing denaturation solution was applied for 7 minutes at 37°C, and it was then left in the lysing solution (Triton X-100, dithiothreitol) for 15 minutes. The samples were gently dehydrated by immersing them in a graded variety of alcohol solutions (70%, 90%, and 100%). After that, the slides were given a five-minute rinse with distilled water. The slides were dried and stained for ten minutes with C, D, and E staining solutions. Finally, the samples were evaluated under Light microscopy. Based on the size and existence of a halo surrounding the nucleus, five distinct cell types were identified to analyze the range of DNA fragmentation. The reported data included the mean percentage of spermatozoa containing non-fragmented DNA (i.e., sperm nucleus DNA with large and medium halo) and fragmented DAN (i.e., sperm nucleus DNA with small halo, without a halo, and cell degradation).

6. Statistical Analysis

PRISM version 9 and IBM SPSS version. 20.0 were used to conduct the statistical analysis (IBM SPSS Corp., Armonk, NY, USA). Continuous variables were expressed as mean ± SD, while categorical variables were expressed as a number (percentage). The Kolmogorov-Smirnov test verified the normality of the variables. If the variables showed normal distribution or were parametric, the relationship between them was assessed using an independent t-test; otherwise, the Man Withney test was used if the variables were non-parametric. The specificity, sensitivity, and cut-off values for each test were ascertained through receiver operating characteristic (ROC) curve analysis, and maximum sensitivity and specificity was used to calculate the cut of point. the Spearman test was employed to assess the correlations between the methods. The P<0.05 was considered as statistically significant.

## Results

**Figure-3:**
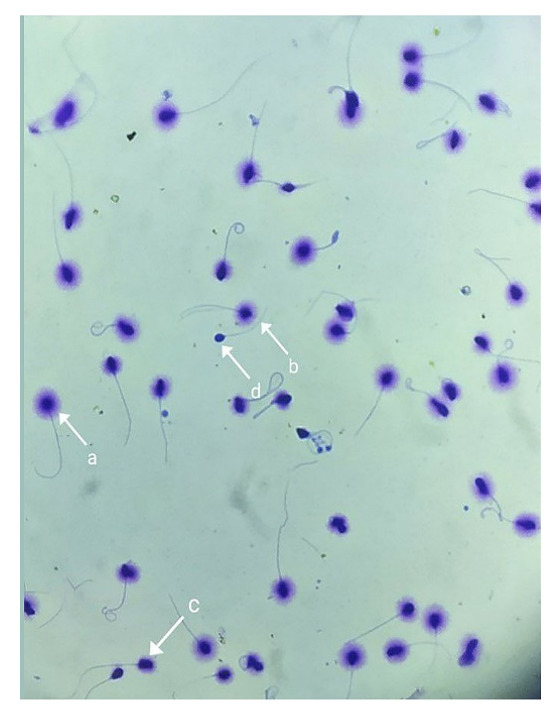
Figure 3. Sperm chromatin dispersion test (SCD) assay under a light microscope was used to determine the integrity of human sperm DNA (Magnification ×1,000). a and b: Defragmented spermatozoa with a large or medium halo. c and d: Fragmented spermatozoa with a low or without the halo.

**Figure-4:**
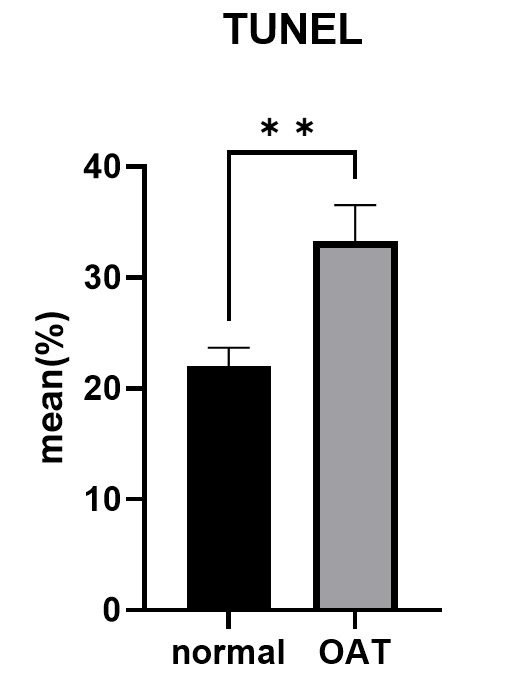
Figure 4. TUNEL levels in the OAT group and the normal group were compared. The mean percentage of TUNEL assay was significantly increased in the OAT group compared to the normal group **: P=0.004.

**Figure-5:**
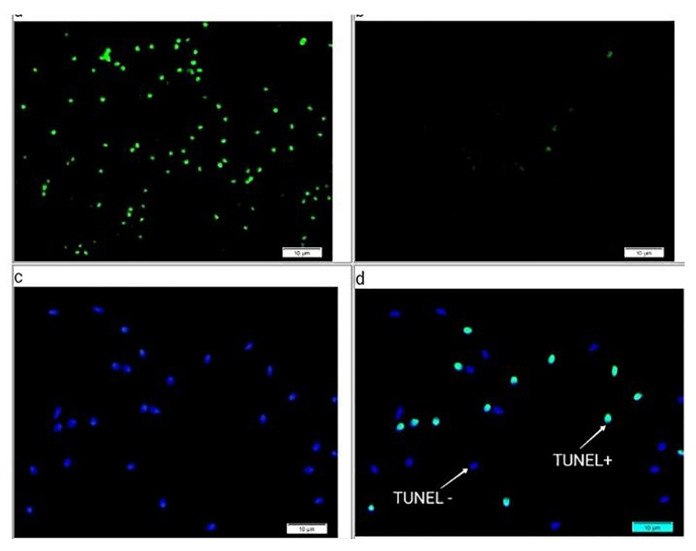
Figure 5. Human SDF assessed by the TUNEL test. The TUNEL assay micrograph identifies sperm with DNA damage (green), and DAPI (blue) staining indicates the total number of nuclei. The TUNEL assay's correct labeling is confirmed by the positive control that was stained following DNAse treatment and the negative control that was not stained. scale bar: 10 μm. positive control (a), negative control (b), DAPI (c), and TUNEL (d).

**Table:** Table3. Specificity, Sensitivity and Cut-off Values Associated with every Assay

**Technique**	**AUC**	**P-value ^1^**	**Cut-off**	**Specificity**	**Sensitivity**
**SCD**	0.71	0.023	19.00	0.90	0.40
**TUNEL**	0.75	0.008	29.00	0.90	0.55

**TUNEL:** terminal deoxynucleotidyl transferase dUTP nick end labelling ; **SCD:** sperm chromatin dispersion;**AUC:** Area under the receiver operating characteristic curve

1. Semen Analysis

The results of sperm parameters including concentration, volume, motility, and morphology achieved from the normozoospermia and OAT men are shown in Table-1. There was a significant difference between two groups in terms of sperm count, progressive motility and morphology of sperms (P<0.001).

2. Malondialdehyde (MDA) Assessment

Seminal levels of MDA were significantly higher in the OAT group (P=0.002, Figure-1) than that of control group. The higher levels of MDA in the present study indicates a higher oxidative stress status of OAT semen. No correlation was found between the mean percentage of typical morphological spermatozoa and the seminal levels of MDA (P=0.832, r=-0.035, Table-2).

3. Sperm DNA Fragmentation

3.1. SCD Test Assay

The results of SCD assay results showed a negative correlation between sperm progressive motility and DFI (P=0.020, r=-0.367, Table-2). Figure-2 illustrates the comparison of SCD between the OAT group and the normal group. Even so, the SCD was significantly increased in the OAT group (P=0.015, P<0.05) compared to the normal group. In addition, the SCD in the two groups was discovered to have a positive correlation with MDA (r=0.232, P=0.149, Table-2). The SDF demonstrated by the Halosperm technique is presented in Figure-3.

3.2. TUNEL Assay

The results of TUNEL assay results demonstrated a negative relationship between the concentration of sperm and DFI (r=-0.335, P=0.034; Table-2). Figure-4 illustrates the comparison between TUNEL in the OAT group and the normal group. While the TUNEL level was considerably increased in the OAT group compared to the normal group (P=0.004). In addition, it was discovered that the TUNEL findings in two groups positively correlated with MDA (r=0.072, P=0.658; Table-2). SDF demonstrated by the TUNEL assay is represented in (Figure-5).

4. Relationships among Procedures

Strong and significant correlations between the SCD test and the TUNEL assay were revealed by Spearman correlation analysis (P=0.008, r=0.739).

4.1. Specificity, Sensitivity, Cut-off values, and ROC Analysis

The specificity, sensitivity and cut-off values of the two distinct assays for predicting OAT men were evaluated using the ROC curve analysis.

**Figure-6:**
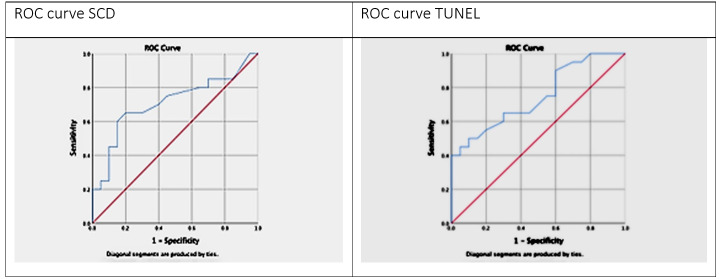
Figure 6. The sperm DNA fragmentation assays' receiver operating characteristic curve

The TUNEL assay showed a larger area under the graph of 0.75, with an SDF cut-off that was29.00 yielding a specificity and sensitivity of 0.90 and 0.55, respectively. The area under the curve of 0.71 for the SCD test with an SDF cut-off value of 19.00 yields a specificity and sensitivity of 0.90 and 0.40, respectively. (Table-3, Figure-6).

## Discussion

Three main topics including the spermatic parameters, the MDA levels of seminal plasma, and sensitivity and efficiency of the TUNEL and SCD assays as two distinct methods for SDF analysis in patients with OAT were evaluated. The sperm count, their morphology, and their progressive motility differed significantly between the two groups. Additionally, a noteworthy inverse relationship was observed between the average spermatozoa percentage and the fragmented DNA, the mean percentage of motile spermatozoa, and sperm concentration. In agreement with these results, a negative correlation between the sperm DFI level, the sperm survival rate, and progressive motility has been previously reported [21][22]. Numerous lipid peroxides that produce ROS target the sperm cell membrane, shattering and destroying the integrity of the sperm DNA strand. According to our findings, the OAT group's seminal plasma MDA levels were significantly greater than that of the normal group. Our results also showed that a high percentage of SDF, highlighted by TUNEL assay and SCD test, appears to be associated with a significant increase in seminal MDA level. Patients with higher sperm DNA damage has been shown to have higher seminal MDA levels[23]. Similar to our study, it has been recently shown that analysis of SDF revealed a significant statistical difference for the detection of DNA fragmentation between normozoospermia and OAT patients in the TUNEL assay and SCD test [24][25][26][27][28] Numerous methods have been created for evaluating SDF with direct clinical applications. While the ideal method for measuring SDF and its thresholds remain to be yet established, the four main SDF tests (Comet, SCD, TUNEL, and SCSA) provide trustworthy data about SDF in subfertility[29][30][31]. However, it is crucial to comprehend how each test presents findings. Numerous studies have reported different clinical values using these methods; however, only the correlation between SCD, TUNEL, and SCSA assays has rarely been proved [32].

We have not only compared the efficiency of TUNEL and SCD assays and their sensitivity to detect SDF, but also the values of the sperm DNA fragmentation index (DFI) reported. The TUNEL technique yielded statistically significant higher estimates of SDF in OAT patients when compared to the SCD test, suggesting that the TUNEL technique has a higher sensitivity in detecting fragmentation of sperm DNA. In fact, our results showed the TUNEL method shows more predictive method than the SCD test for the detection of DNA fragmentation in OAT patients. The ROC curve showed that the TUNEL assay was more sensitive than the SCD test for the detection of DNA fragmentation in OAT patients (Table-3, Figure-6). On the other hand, the TUNEL test with an area under the curve of 0.745 and an SDF threshold of 29.00 showed higher sensitivity and specificity. These results are almost exact replicas of those obtained by Javed et al. [20] who has reported a sensitivity, specificity, and area under the curve of 0.754, 0.942, and 0.901, respectively, with a cut-off estimation of 22.08%. Our findings are in line with previous research that has reported values of roughly 20%, but our estimated limit of 19.00% for SCD is on the low end of the distribution. In order to ascertain sensitivity and specificity, Ribas Maynou et al. also used receiver operating characteristic curves[32]. According to their findings, the alkaline comet assay was the most reliable technique for identifying DNA fragmentation in infertile males. It was followed by the neutral comet assay, TUNEL assay, SCD test, and SCSA.

Nonetheless, infertile patients dependably have showed a high SDF[20]. Both of the methods' dependability in evaluating sperm DNA fragmentation is confirmed by our results, which align with earlier research. These results suggest that different methods may detect different aspects of SDF, since the TUNEL assay directly detects DNA fragmentation and SCD focuses on chromatin fragmentation. Thus, in the bimodal distribution among OAT participants, the cut-off SDF value demonstrated low specificity and considerable sensitivity, as has been previously reported. Clinical data from two widely used techniques that the majority of frequently utilized to evaluate SDF in a similar collection of patients are presented in this study. These findings suggest that two methods are useful in distinguishing between OAT patients and fertile individuals; however, the TUNEL assay is a better predictor of OAT patients than the SCD test.

## Conclusion

According the results of the current study the OAT patients show higher levels of seminal plasma level of MDA and DNA fragmentation. It seems that sperm DNA fragmentation can be detected with high efficiency and sensitivity using both TUNEL and SCD assays. However, the TUNEL test was found to be a more accurate predictor for OAT patients

## Acknowledgment

We are grateful to every patient who consented to take part in this research. We appreciate the support of all of the clinical staff at Arash Women's Hospital's infertility department.

## Conflict of Interest

The author(s) declared no potential conflicts of interest with respect to the research, authorship, and/or publication of this article.

## References

[R1] Organization WH (2023).

[R2] Colpi GM, Francavilla S, Haidl G, Link K, Behre HM, Goulis DG (2018). European Academy of Andrology guideline Management of
oligo-astheno-teratozoospermia. Andrology.

[R3] Azad N, Nazarian H, Ghaffari Novin, Masteri Farahani, Piryaei A, Heidari MH, Abdollahpour Alitappeh (2018). Oligoasthenoteratozoospermic (OAT) men display altered
phospholipase C ζ
(PLCζ) localization and a lower percentage of sperm cells expressing PLCζ
and
post-acrosomal sheath WW domain-binding protein (PAWP). BJBMS.

[R4] Wei X, Liu W, Zhu X, Li Y, Zhang X, Chen J (2021). Biallelic mutations in KATNAL2 cause male infertility due to
oligo-astheno-teratozoospermia. Clinical genetics.

[R5] Alahmar AT (2023). The Effect of Selenium Therapy on Semen Parameters, Antioxidant
Capacity,
and Sperm DNA Fragmentation in Men with Idiopathic Oligoasthenoteratospermia. Biol Trace Elem Res.

[R6] Ribeiro S, Sharma R, Gupta S, Cakar Z, De Geyter, Agarwal A (2017). Inter- and intra-laboratory standardization of TUNEL assay for
assessment
of sperm DNA fragmentation. Andrology.

[R7] Candela L, Boeri L, Capogrosso P, Cazzaniga W, Pozzi E, Belladelli F (2021). Correlation among isolated teratozoospermia, sperm DNA
fragmentation and
markers of systemic inflammation in primary infertile men. PloS one.

[R8] Bahrami Z, Daeifarshbaf N, Amjadi F, Aflatoonian R (2022). The effects of hormonal changes on sperm DNA integrity in
oligoasthenoteratospermia individuals: A case-control study. IJRM.

[R9] Yang H, Li G, Jin H, Guo Y, Sun Y (2019). The effect of sperm DNA fragmentation index on assisted
reproductive
technology outcomes and its relationship with semen parameters and lifestyle. TAU.

[R10] Tamburrino L, Marchiani S, Montoya M, Elia Marino, Natali I, Cambi M (2012). Mechanisms and clinical correlates of sperm DNA damage. AJA.

[R11] Agarwal A, Panner Selvam, Baskaran S, Cho CL (2019). Sperm DNA damage and its impact on male reproductive health: a
critical
review for clinicians, reproductive professionals and researchers. Expert Rev.Mol.Diagn.

[R12] Yan B, Ye W, Wang J, Jia S, Gu X, Hu H (2022). Evaluation of Sperm DNA Integrity by Mean Number of Sperm DNA
Breaks
Rather Than Sperm DNA Fragmentation Index. Clinical chemistry.

[R13] Agarwal A, Majzoub A, Baskaran S, Panner Selvam, Cho CL, Henkel R (2020). Sperm DNA Fragmentation: A New Guideline for Clinicians. The world journal of men's health.

[R14] Hamilton TRdS, Assumpção MEODÁ (2020). Sperm DNA fragmentation: causes and identification. Zygote (Cambridge, England).

[R15] Esteves SC, Gosálvez J, López-Fernández C, Núñez-Calonge R, Caballero P, Agarwal A, Fernández JL (2015). Diagnostic accuracy of sperm DNA degradation index (DDSi) as a
potential
noninvasive biomarker to identify men with varicocele-associated infertility. International urology and nephrology.

[R16] Sharma R, Martinez MP, Agarwal A (Male). Sperm Chromatin Integrity Tests and Indications. In: Parekattil SJ, Esteves SC, Agarwal A, editors.

[R17] Ribas-Maynou J, Benet J (2019). Single and Double Strand Sperm DNA Damage: Different Reproductive
Effects
on Male Fertility. Genes.

[R18] Baskaran S, Agarwal A, Panner Selvam, Finelli R, Robert KA, Iovine C (2019). Tracking research trends and hotspots in sperm DNA fragmentation
testing
for the evaluation of male infertility: a scientometric analysis. Reproductive biology and endocrinology : RBE.

[R19] Simon L, Emery BR, Carrell DT (2017). Review: Diagnosis and impact of sperm DNA alterations in assisted
reproduction. Best practice & research Clinical obstetrics & gynaecology.

[R20] Javed A, Talkad MS, Ramaiah MK (2019). Evaluation of sperm DNA fragmentation using multiple methods: a
comparison of their predictive power for male infertility. Clin Exp Reprod Med.

[R21] Ferrigno A, Ruvolo G, Capra G, Serra N, Bosco L (2021). Correlation between the DNA fragmentation index (DFI) and sperm
morphology of infertile patients. Journal of assisted reproduction and genetics.

[R22] Chua SC, Yovich SJ, Hinchliffe PM, Yovich JL (2023). How Well Do Semen Analysis Parameters Correlate with Sperm DNA
Fragmentation A Retrospective Study from 2567 Semen Samples Analyzed by the
Halosperm Test. Journal of Personalized Medicine.

[R23] Liu K, Mao X, Pan F, Chen Y, An R (2023). Correlation analysis of sperm DNA fragmentation index with semen
parameters and the effect of sperm DFI on outcomes of ART. Scientific Reports.

[R24] Kumalic SI, Klun IV, Bokal EV, Pinter B (2020). Effect of the oral intake of astaxanthin on semen parameters in
patients
with oligo-astheno-teratozoospermia: a randomized double-blind
placebo-controlled
trial. Radiology and oncology.

[R25] Kooshesh L, Bahmanpour S, Zeighami S, Nasr-Esfahani MH (2020). Effect of Letrozole on sperm parameters, chromatin status and ROS
level
in idiopathic Oligo/Astheno/Teratozoospermia. Reproductive biology and endocrinology : RBE.

[R26] Huang WJ, Lu XL, Li JT, Zhang JM (2020). Effects of folic acid on oligozoospermia with MTHFR polymorphisms
in term
of seminal parameters, DNA fragmentation, and live birth rate: a
double-blind,
randomized, placebo-controlled trial. Andrology.

[R27] Micic S, Lalic N, Djordjevic D, Bojanic N, Bogavac-Stanojevic N, Busetto GM (2019). Double-blind, randomised, placebo-controlled trial on the effect
of
L-carnitine and L-acetylcarnitine on sperm parameters in men with idiopathic
oligoasthenozoospermia. Andrologia.

[R28] Campos LGA, Requejo LC, Miñano CAR, Orrego JD, Loyaga EC, Cornejo LG (2021). Correlation between sperm DNA fragmentation index and semen
parameters in
418 men seen at a fertility center. JBRA assisted reproduction.

[R29] Martinez M, Majzoub A (2021). Best laboratory practices and therapeutic interventions to reduce
sperm
DNA damage. Andrologia.

[R30] Henkel R, Hoogendijk CF, Bouic PJ, Kruger TF (2010). TUNEL assay and SCSA determine different aspects of sperm DNA
damage. Andrologia.

[R31] Esteves SC, Agarwal A, Majzoub A (2017). The complex nature of the sperm DNA damage process. Translational andrology and urology.

[R32] Ribas-Maynou J, García-Peiró A, Fernández-Encinas A, Abad C, Amengual MJ, Prada E (2013). Comprehensive analysis of sperm DNA fragmentation by five
different
assays: TUNEL assay, SCSA, SCD test and alkaline and neutral Comet assay. Andrology.

